# PEG Gels Significantly Improve the Storage Stability of Nucleic Acid Preparations

**DOI:** 10.3390/gels8120819

**Published:** 2022-12-12

**Authors:** Pengfei Cui, Luping Ma, Pengju Jiang, Cheng Wang, Jianhao Wang

**Affiliations:** School of Pharmacy, Changzhou University, Changzhou 213164, China

**Keywords:** nucleic acid preparation, PEG gelation, stability

## Abstract

Currently, nucleic acid preparations have gained much attention due to their unique working principle and application value. However, as macromolecular drugs, nucleic acid preparations have complex construction and poor stability. The current methods to promote stability face problems such as high cost and inconvenient operatios. In this study, the hydrophilic pharmaceutical excipient PEG was used to gelate nucleic acid preparations to avoid the random movements of liquid particles. The results showed that PEG gelation significantly improved the stability of PEI25K−based and liposome−based nucleic acid preparations, compared with nucleic acid preparations without PEG gelation. After being stored at 4 °C for 3 days, non−PEG gelled nucleic acid preparations almost lost transfection activity, while PEGylated preparations still maintained high transfection efficiency. Fluorescence experiments showed that this effect was caused by inhibiting particle aggregation. The method described in this study was simple and effective, and the materials used had good biocompatibility. It is believed that this study will contribute to the better development of gene therapy drugs.

## 1. Introduction

Nucleic acid preparations, including DNA and RNA preparations, play an increasingly important role in today’s biopharmaceutical industry [1]. For example, the mRNA vaccine preparation has been widely used in the current COVID−19 epidemic, greatly reducing the fatality rate and easing people’s panic [2,3,4]. Another classic application in DNA preparation is CAR−T [5,6,7]. The so−called CAR−T employs viruses to load functional DNA and then transform immune cells. Finally, the transformed cells are reinfused to the patient for immunotherapy. Currently, CAR−T has brought hope to patients with acute lymphoblastic leukemia, diffuse large B−cell lymphoma, acute myeloid leukemia, etc. [8,9].

Nucleic acid preparations have made great contributions to maintaining the health of people. However, they also involve some problems that need to be overcome. For example, mRNA is unstable, and nucleic acid products are susceptible to degradation. Therefore, the transportation of mRNA vaccines requires certain conditions, such as ultra−low−temperature cold−chain transportation [10,11], bringing a heavy economic burden to less developed regions. Moreover, researchers have proposed some measures to improve the stability of mRNA vaccines, including adding new excipients, improving prescriptions, or making freeze−dried powder injections [12]. These methods improve the stability of mRNA vaccines to a certain extent, but additional costs are caused by the need for compatibility testing for changes in formulation and process. For CAR−T, the treatment process is complicated, and the cost is high. An important step in the process of CAR−T therapy is the transfection of isolated cells [13,14]. Therapeutic genes are loaded by viruses formed into nanoparticles, and then introduced into cells. This step requires special environments and professional operation which limits its large−scale application [15]. 

**Figure 1:**
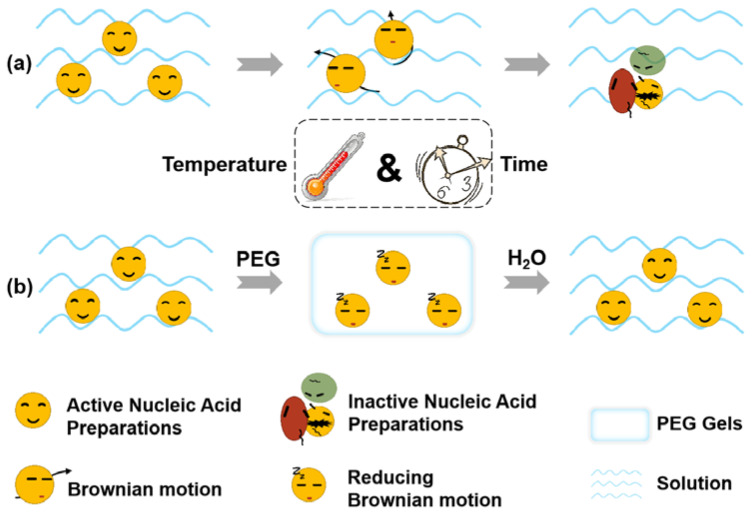
Scheme of improving the storage stability of nucleic acid preparations by PEG gelation. (**a**) Formation of aggregates by the collision of nucleic acid preparations in solution through Brownian motion with activity loss. (**b**) When the nucleic acid preparations are wrapped by PEG, the displacement of the nucleic acid particles is decreased and the stability is increased. After adding the aqueous solvent, the active state of the nucleic acid preparations is restored.

One of the main reasons why nucleic acid preparations need to be stored at ultra−low temperatures or need complicated operations before use is the instability of nucleic acids preparations [16]. There are many factors that affect the stability of nucleic acid preparations, such as aggregation, adsorption, chemical reaction, freeze−thaw, shear force, and temperature. Usually, nucleic acids are presented as nanoparticles with viral or non−viral vectors. Nanoparticles have a large specific surface area, with almost all atoms concentrated on the particle surface in a highly activated state, thus resulting in the insufficient coordination number of surface atoms and high surface energy. Therefore, nanoparticles often encounter together to form aggregates by Brownian motion (Figure 1a). However, nucleic acid drugs act by releasing nucleic acids in the cytoplasm or nucleus. The aggregation of nucleic acid nanoparticles may limit the release of nucleic acid, thus affecting the efficacy. By taking spherical particles as the model, Einstein deduced the Einstein−Brownian motion formula as below [17].

(1) $$\Delta =\sqrt{\frac{RTt}{L3\pi \eta r}}$$

As shown in Formula (1), particle displacement (Δ) in sol is related to particle size (*r*), medium viscosity (*η*), temperature (*T*), and observation time (*t*). The displacement can be decreased by lowering the temperature (*T*) or increasing the medium viscosity (*η*). In other words, the stability of nucleic acid preparations can be improved by reducing the temperature or increasing the viscosity. For example, nucleic acid solutions, including mRNA vaccines, are stored in an ultra−low−temperature environment. Furthermore, some companies have developed the freeze−drying process. However, these measures require special equipment or increase the complexity of the formulation.

Poly (ethylene glycol) (PEG) hydrogels have been widely used as drug delivery matrices over the last few decades [18,19]. Because of its versatility and excellent biocompatibility, PEG macromer chemistry has promoted the development of numerous intelligently designed hydrogel systems [20]. For example, by using PEG as a matrix suppository, irregular movements of drugs in water can be avoided, while drugs can also be released quickly after contacting the water [21,22,23]. Inspired by the suppository prescription, PEG was employed to solidify the nucleic acid preparation particles in this work, so that the viscosity index η could increase infinitely, thus reducing the Brownian motion displacement in solution. PEI25K (PEI), a commonly used non−viral vector, was adopted, aiming at complicating plasmid DNA to form DNA preparations [24]. By adding a water−based solvent before use, the gelled PEI/DNA nanoparticles can be release to the liquid in a short time (Figure 1b). It is believed that this method can provide a promising direction for the development of nucleic acid preparations.

## 2. Results and Discussion

### 2.1. Synthesis of PEI/DNA@PEG Systems

PEG is widely used for drug delivery, including nanoparticle delivery [25] and matrices for suppositories [26]. In this study, the process of suppository preparation was simulated, aiming at making nucleic acid preparations [27]. The main preparation methods of suppositories are the fusion method and the cold compressing method. For nucleic acid preparations in a liquid state, the fusion method is more suitable. PEG with a suitable melting point was selected for this work, specifically, PEG in a solid state at room temperature and melting quickly into a liquid at 60 °C. Moreover, PEI, a commonly used non−viral gene−delivery system, was also employed to form nucleic acid preparations in this research.

**Figure 2:**
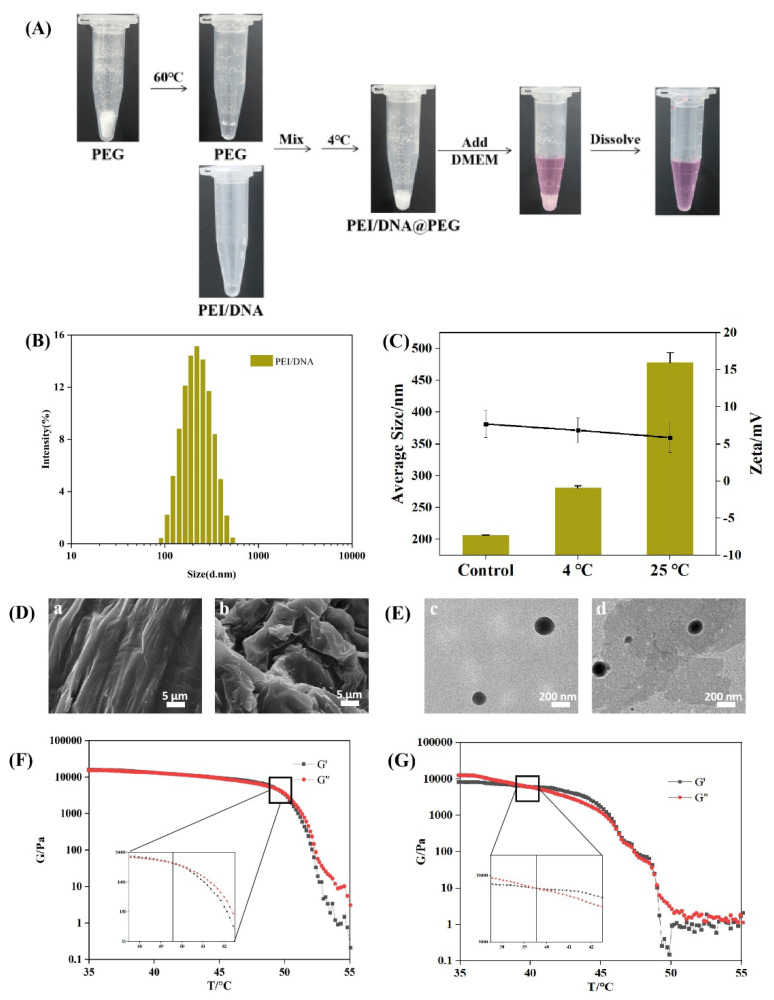
Synthesis and characterization of PEI/DNA@PEG. (**A**) Preparation process of gelled PEI/DNA (PEI/DNA@PEG). (**B**) Particle size of PEI/DNA complex. (**C**) Size stability of PEI/DNA nanoparticles at 4 °C and 25 °C for 3 days was determined by DLS. (*n* = 3) (**D**) SEM of (**a**) PEG, (**b**) PEI/DNA@PEG. (**E**)TEM of (**c**) PEI/DNA. (**d**) PEI/DNA@PEG (aqueous solution). Melting point of PEG (**F**) and PEI/DNA@PEG (**G**).

The microstructure of freeze−dried PEG and PEI/DNA@PEG gel was observed using SEM. It was found that the surface of PEG gel was relatively smooth, while the surface of PEI/DNA@PEG gel had more pores (Figure 2D). PEI complexed with plasmid DNA to form PEI/DNA nanoparticles in solution through electrostatic interactions according to the reported method [28]. However, the nanoparticles stored for 3 days will partially aggregate, resulting in a larger particle size (Figure 2C).

A similar sphere can be clearly seen from the TEM image of the sample. There was no obvious aggregation between the particles. The dispersion was good. The particle size distribution of the micelles was narrow. The average particle size of PEI/DNA nanoparticles was 205.0 ± 10.26 nm (Figure 2B). Most of the PEI/DNA released after PEG storage showed relatively uniform spherical particles (Figure 2E). It can also be seen by measuring the melting point that the melting point of PEG gel was about 49.6 °C. After adding PEI/DNA nanoparticles, the melting point of the gel decreased to 39.6 °C (Figure 2F,G).

As shown in Figure 2A, the melting PEG liquid and PEI/DNA solution were mixed and kept at 4 °C, thereby obtaining homogeneous gelled PEI/DNA (PEI/DNA@PEG). When adding DMEM cell culture medium or any other water—based solvent, the gels can be dissolved quickly before applying to cells. The manufacturing process of nucleic acid gels is very easy, and the operating procedures before use are also very simple.

### 2.2. Combination of PAA and PEI in PEG System

**Figure 3:**
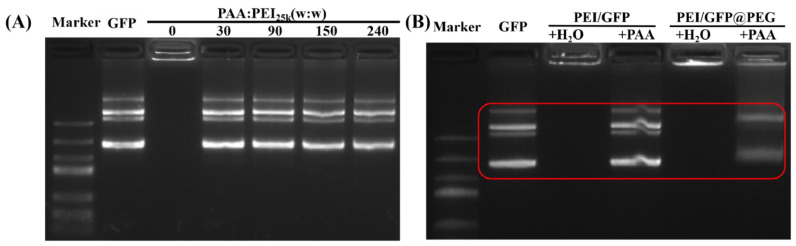
(**A**) Combination of PAA with PEI in different mass ratios to release GFP. (**B**) PAA combined with PEI to release GFP in PEG gel system.

The release of nucleic acids is one of the key factors that affects the efficacy of nucleic acid preparations [29,30]. To verify whether PEG encapsulation affects gene release, polyacrylic acid (PAA) was employed in gel electrophoresis experiments. As an anionic polymer in water, PAA could competitively replace the negatively charged GFP from the PEI/GFP complex (Figure 3A,B), thereby achieving the purpose of releasing GFP [31]. In detail, PEI/GFP@PEG was first dissolved in DMEM cell culture medium, and then incubated with PAA. As shown in Figure 3B, white bands were observed in the PAA—added lane, indicating the release of GFP from PEI/GFP@PEG. However, the white GFP bands of PAA—added PEI/GFP@PEG were lighter than that of the control group, indicating that PEG had some effect on the binding of PEI/GFP, which may be attributed to the non—covalent interaction between the PEG polymers.

### 2.3. In Vitro Safety Assay of PEI/DNA@PEG and Lipo/DNA@PEG

**Figure 4:**
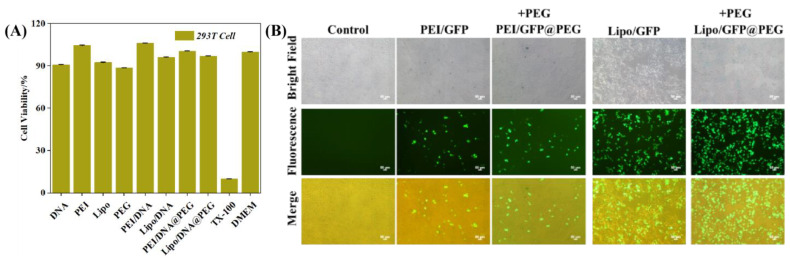
(**A**) The cytotoxicity of PEI/DNA@PEG and Lipo/DNA@PEI against 293T (*n* = 5). (**B**) 24 h images of transfected cells after being treated with PEI/GFP, PEI/GFP@PEG, Lipo/GFP, and Lipo/GFP@PEG. The addition of PEG does not destroy the release of GFP.

The biocompatibility of PEG gel was determined by in vitro cytotoxicity experiments. The activity of 293T cells reached more than 90%, indicating that PEG gel had good biological safety (Figure 4A).

### 2.4. Transfection Experiments of the System

Cell transfection efficacy of the nucleic acid preparation PEI/DNA@PEG was evaluated using 293T cells. GFP plasmid was employed here to indicate the transfection efficacy. PEI/GFP@PEG was obtained by adopting the methods described in Figure 2A. After that, PEI/GFP@PEG was dissolved in DMEM cell culture medium, and then applied to cells. As indicated in Figure 4B, 15.3% of cells were green—fluorescence positive in the PEI/GFP treated group, while 13.7% of cells were transfected in the PEI/GFP@PEG treated group, which means the PEG—gelled nucleic acid preparation PEI/GFP@PEG had a similar transfection efficiency to PEI/GFP alone. This result gives us great encouragement. In order to verify the wider application of the strategy, a commercial liposome—based transfection reagent was employed. Therefore, Lipo/GFP@PEG was prepared with the methods described in Figure 2A. As shown in Figure 4B, PEG gelation did not affect the transfection of the liposome—based transfection reagent. Lipo/GFP@PEG (37.2% GFP positive cells) had a similar performance to Lipo/GFP (39.5% GFP positive cells) during transfection.

### 2.5. Long—Term Transfection Experiments

**Figure 5:**
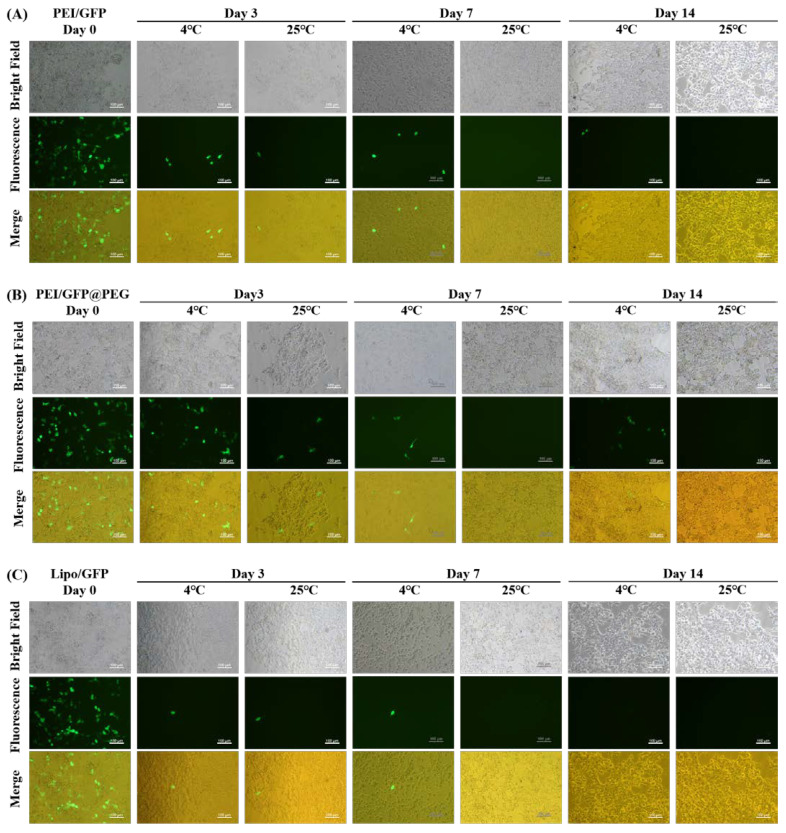

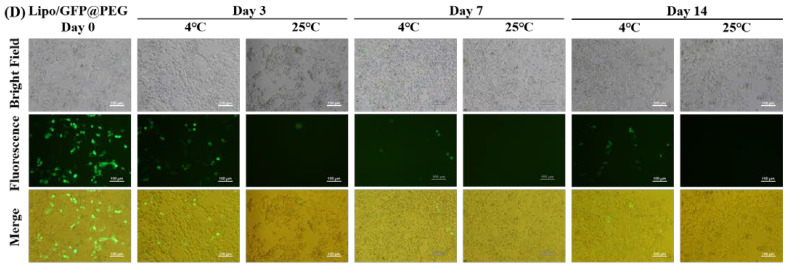
Transfection stability assay of 293T cells treated with PEI/GFP (**A**), PEI/GFP@PEG (**B**), Lipo/GFP (**C**), and Lipo/GFP@PEG (**D**).

**Figure 6:**
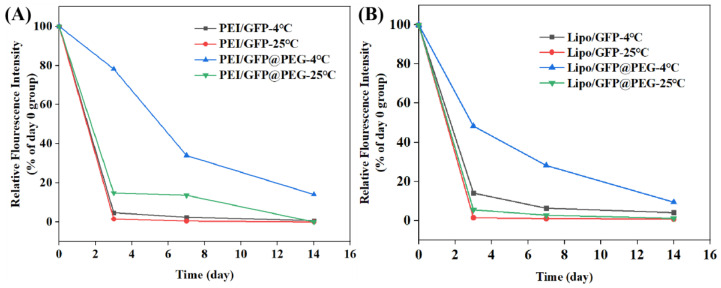
Flow quantitative results of 293T cell transfection with PEI/GFP, PEI/GFP@PEG (**A**), and Lipo/GFP, Lipo/GFP@PEG (**B**).

Dissolving gene preparations immediately after PEG gelation does not affect the transfection efficiency. Thus, to test how long this effect can be sustained, gelled GFP products were prepared and stored at 4 °C and 25 °C. The transfection efficiencies of gelled GFP preparations were assayed using 293T on day 0, day 3, day 7, and day 14. In addition, non—gelled preparations of PEI/GFP and Lipo/GFP were performed as control groups in the manner described above. Transfected cell images of different groups are shown in Figure 5. Transfection efficiency of non—gelled preparations drops sharply on day 3, while that of gelled preparations was maintained at a certain level. In order to obtain more accurate data, cells were collected, and the fluorescence intensities of the cells were determined with a flow cytometer (C6, BD Biosciences). Results are shown in Figure 6. In contrast with the day 0 group, nearly 80% fluorescence intensity of PEI/GFP@PEG was retained after 3 days of storage at 4 °C, while only 4.7% fluorescence intensity of PEI/GFP was observed. When the storage temperature rose to 25 °C, both groups exhibited worse conditions. Nevertheless, PEI/GFP@PEG kept a higher fluorescence than PEI/GFP (14.8% vs. 1.4%, respectively). Similar contrasts can be seen between Lipo/GFP and Lipo/GFP@PEG (Figure 6B). DNA is a hydrophilic macromolecule, and there are not many DNA enzymes in the air, so the DNA aqueous solution is relatively stable. However, the transfection of DNA requires the assistance of a carrier. When DNA is encapsulated into the gene carrier to form nanoparticles, nanoparticles may sediment due to the irregular movement of nanoparticles in aqueous solution. Successful DNA transfection requires not only the encapsulation and protection of the carrier, but also its successful entry into the cell for DNA release. Also, the sedimentation of the particles may interfere with the release of DNA [32,33]. Nano—scale particles have a high surface area, and the particles are easier to aggregate. Therefore, during cell transfection in vitro, the aggregated particles cannot release genes in a timely manner, resulting in a low transfection efficiency [34,35]. In contrast, in this study, the gene complexes were within the PEG gel, thereby restricting the random motion of the particles. When adding water to the system to dissolve, the whole system could still continue to release the non—aggregated state of the gene complex particles, thus realizing transfection.

### 2.6. Combination of Calcein and PEI in PEG System

**Figure 7:**
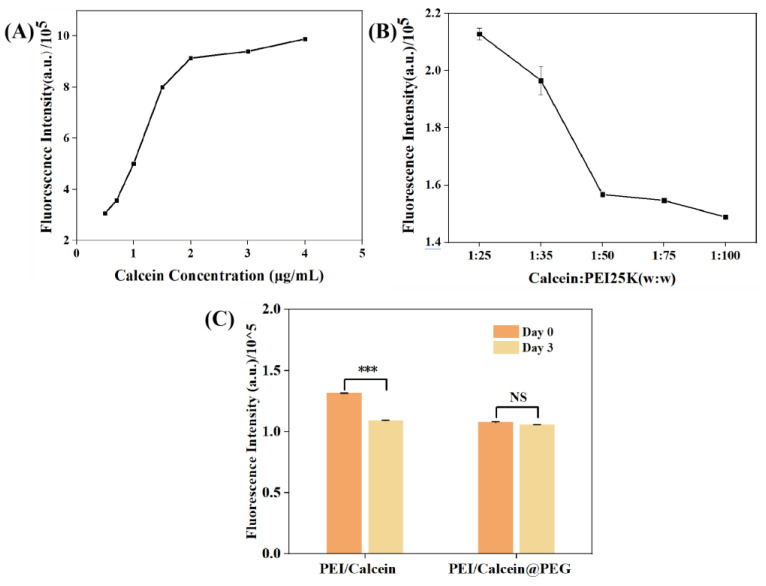
(**A**) Relationship between Calcein concentration and fluorescence intensity. (**B**) Fluorescence values of PEI/Calcein complex at different ratios. (**C**) Fluorescence intensity of PEI/Calcein and PEI/Calcein@PEG, Calcein: PEI = 1:50 (*w*:*w*), *n* = 3, *** *p* < 0.001, not significant (NS).

The higher transfection efficiency of PEG—gelled gene complexes is caused by the immobilization, which further limits particle aggregation. To demonstrate this, measurement of the change in particle size of the gene complexes before and after gelation was first considered. However, unfortunately, as there were numerous PEG molecules in water, the measurement of hydrated particle size was hindered (data not shown). This can be applied to the aggregation of PEG in water. Although macroscopically PEG is water−soluble, the repeating unit of PEG is amphiphilic, so the particle size can be measured with a light scattering particle size analyzer. To solve this problem, a fluorescent dye, called Calcein, was employed [36]. Calcein is an anionic fluorescein molecule with excitation light wavelength of 495 nm and emission light wavelength of 515 nm [37]. Its molecular weight is greater than 500, and each molecule has four carboxyl groups, able to replace DNA to form a complex with PEI partly. Normally, the fluorescence efficiency may drop significantly when the fluorescent molecules are converted from the dispersed state to the aggregated state [38]. Calcein was used to simulate the binding of DNA to PEI. The concentration of Calcein was positively correlated with the fluorescence intensity (Figure 7A). An amount of 1 ug/mL of Calcein was selected to form the complex with PEI. However, the fluorescence intensity decreased with the increase in PEI concentration, proving that PEI did form aggregates with Calcein (Figure 7B). Afterwards, PEI/Calcein@PEG was prepared and stored at 4 °C. After 3 days, both PEI/Calcein and PEI/Calcein@PEG were taken out, and the fluorescence intensity was determined with a fluorescence spectrometer. As presented in Figure 7C, the fluorescence intensity of the PEI/Calcein complex decreased significantly after 3 days of storage at 4 °C. However, the fluorescence emission intensity of gelled PEI/Calcein@PEG barely changed after 3 days of storage. From the change in fluorescence value, it could be inferred that PEI/Calcein in aqueous solution had aggregated, while gelled PEI/Calcein@PEG had not aggregated, indicating the aggregation of PEI/Calcein nanoparticles in solution. This phenomenon was consistent with the hypothesis that gelation could improve the stability of nanoparticles which was beneficial for nucleic acid preparations.

## 3. Conclusions

In summary, in view of the instability of gene drugs, the commonly used pharmaceutical excipient PEG was employed to gel the gene drugs using the melting method, thereby avoiding the aggregation failure caused by Brownian motion of the gene drug particles in the solution state. As demonstrated in this study, PEG gelation did not affect the release of gene drugs. Cell transfection and storage experiments proved that the PEG−gelled gene drug could maintain the better transfection activity. Through fluorescence aggregation experiments, it was verified that PEG gelation can inhibit the aggregation effect of particles. The method described in this study used simple and safe materials, could offer rapid preparation and good effect, and had great application potential in many fields such as cell transfection and gene therapy. Compared to the newly prepared gene preparation after 14 days of storage, this was unacceptable, either clinically or industrially. What caused the decreased transfection efficiency of gelled gene preparations? As indicated in Figure 3, PAA competed with DNA from PEI/DNA. This demonstrated that PEG gelation did not prevent DNA release. Nevertheless, compared with the nucleic acid preparation solution, PEG gelation significantly improved the gene transfection efficiency; there was still a 90% decrease in efficiency. It was also noticed that the brightness of the released DNA bands was weaker than that of the ungelled PEI/DNA, indicating that PEG had some effect on the binding of PEI/DNA. This might be attributed to the non−covalent interaction between the PEG polymers. Furthermore, future exploration is needed to obtain more efficient gelled nucleic acid preparations and to benefit the development of the nucleic acid drug industry.

## 4. Materials and Methods

### 4.1. Materials

Poly (ethylene glycol) (PEG, molecular weight 2000) was obtained from Huiyou Chemical (Beijing, China). Polyacrylic acid (PAA) was purchased from Aladdin Biochemical (Shanghai, China). Polyethylenimine (PEI, molecular weight 25,000), dimethyl sulfoxide (DMSO), MTT, and deoxyribonucleic acid from herring sperm (DNA) were provided by Sigma−Aldrich (Shanghai, China). Fetal bovine serum (FBS), trypsin, penicillin–streptomycin, and Dulbecco’s modified Eagle’s medium (DMEM) were purchased from Gibco (Grand Island, NE, USA). Lipo8000™ was obtained from Beyotime (Shanghai, China). GFP plasmid was obtained by using E.Z.N.A.^®^ Fastfilter Endo−free Plasmid Maxi kit (Omega, Connecticut, USA). The 293T cells (Human Renal Tubular Epithelial Cells) were from National Collection of Authenticated Cell Cultures (Shanghai, China).

### 4.2. Preparation and Characterization of PEI/DNA@PEG

PEI/DNA complex was prepared following methods described before [39]. In detail, 10 mg PEI and 10 mg DNA were dissolved in 100 mL of distilled water. Therefore, 100 μL of 0.1 mg/mL DNA was slowly added to 100 μL of 0.1 mg/mL PEI solution to form PEI/DNA nanoparticles. Then the particle size and surface charge of PEI/DNA were measured with a Malvern particle size analyzer (Malvern, UK). The stability of the prepared PEI/DNA nanoparticles at 4 °C and 25 °C for 3 days was analyzed.

The PEG (50 mg) was melted into a colorless and transparent liquid in a water bath at 60 °C. Then, the temperature of the water bath was adjusted to 50 °C to keep PEG (50 mg) in the liquid state. A total of 20 μL of PEI/DNA nanoparticles were added to the melted PEG at 50 °C (PEI:DNA:PEG = 1 μg:1 μg:50 mg). After the samples were refrigerated at 4 °C for 5 min, PEI/DNA@PEG gels could be obtained.

At the same time, the microstructure of each group of samples was observed by scanning electron microscopy (SEM, Regulus−8100, Tokyo, Japan) and transmission electron microscopy (TEM, H−7800, Tokyo, Japan). An appropriate amount of PEG and PEI/DNA@PEG samples was placed in a centrifuge tube, sealed with a sealing membrane, and quickly frozen at −80 °C for 2 h. After removal, a number of holes were punctured in the sealing membrane with a 1 mL syringe needle, and the sample was placed in a freeze dryer (Alpha 2−4 LSCbsic, Christ, Osterode, Germany) for freeze−drying. The structural morphology of the two samp \les was observed under an accelerated voltage of 10.0 kV under an SEM. The PEI/DNA and PEI/DNA@PEG aqueous solutions were observed using TEM. Finally, the prepared PEI/DNA@PEG gels and PEG gels were scanned with a rheometer (AR 1500 ex, TA Instruments, New Castle, DE, USA) to measure the melting point of the two gels.

### 4.3. Combination of PAA and PEI in PEG System

Considering the negative charge of PAA, PAA was substituted for GFP in PEI/GFP nanoparticles, thus proving the good storage of GFP. In detail, 2 μL of 0.15 mg/mL GFP was added to 2 μL of 0.15 mg/mL PEI during vortex, and then PEI/GFP nanoparticles were reacted for 20 min. At the end of the reaction, PAA solutions with different mass ratios (GFP:PAA = 1:30,90,150,240; *w*:*w*) were added to the PEI/GFP solution, and fully mixed with a pipette. The sample then reacted for 10 min. Then, the loading buffer was added, and GFP agarose gel electrophoresis was performed.

For the release of GFP in PEI/GFP@PEG gel, the gel was prepared by applying the preparation method of Section 4.2. Then, the gel was dissolved in 450 μL distilled water, and a 37 °C water bath was used to ensure its complete dissolution, followed the addition of PAA solution. After the complete reaction, agarose gel electrophoresis was performed.

### 4.4. In Vitro Safety Assay of PEI/DNA@PEG and Lipo/DNA@PEG

The safety of PEG gel was analyzed with a MTT cytotoxicity test. 293T cells were seeded on a 96−well plate at a density of 10,000 per 200 μL for 18 h. The PEI/GFP@PEG (50 mg PEG) solids and Lipo/GFP@PEG (50 mg PEG) solids prepared above were added to 450 μL of DMEM cell culture medium and placed in a 37 °C water bath to heat and dissolve. The 96−well plate upper medium was discarded. The fully dissolved PEG sample was added to the well plate. After 4 h of incubation, the sample wells were replaced with 100 μL of DMEM cell culture medium. After 24 h, 20 μL of concentrated MTT solution (5 mg·mL^−1^) was added. The supernatants were removed carefully 4 h later, and 150 μL of dimethyl sulfoxide (DMSO) was added to dissolve the crystal violet at the bottom of the well. Finally, absorbance (OD) was measured with a microplate reader (Thermo Scientific, CHN, Waltham, MA, USA) at 570 nm. Cytotoxicity was calculated using the following Equation (2):

Cytotoxicity (%) = OD _test group_/OD _blank control group_ × 100% (2)

### 4.5. Transfection Experiments of the System

293T cells were seeded on a 24−well plate at a density of 150,000 per mL for 18 h. The PEI/GFP@PEG (50 mg PEG) solids and Lipo/GFP@PEG (50 mg PEG) solids prepared above were added to 450 μL of DMEM cell culture medium and placed in a 37 °C water bath for heating and dissolution. The 24−well plate upper medium was discarded. The fully dissolved PEG samples were added to the well plate. After 4 h of incubation, the sample wells were replaced with 1 mL of cell culture medium, and the images were taken after 24 h of incubation using a fluorescence microscope (Eclipse Ti2−U, Nikon, Tokyo, Japan).

### 4.6. Long−Term Transfection Experiments

The PEI/GFP@PEG and Lipo/GFP@PEG solids were stored in the environment of 4 °C and 25 °C for 3 days, 7 days, and 14 days, and then transfection experiments were performed according to Section 4.5.

### 4.7. Combination of Calcein and PEI in PEG System

Calcein at a concentration of 0–5 μg/mL was prepared and its fluorescence value was measured using a fluorescence spectrophotometer (FS5, Edinburgh Instruments). Calcein was mixed with different concentrations (Calcein: PEI = 1:25,35,50,75,100; *w*:*w*) PEI, and their fluorescence values were measured. PEI (PEI: Calcein = 50:1; *w*:*w*) was reacted with Calcein, added to the melted PEG, and left to solidify completely. Afterwards, a PEI/Calcein@PEG sample was obtained, and dissolved after 3 days, with the fluorescence value measured.
